# Treatment of hip instability after total hip replacement with pelvic osteotomy using a modified Stoppa approach

**DOI:** 10.1016/j.ijscr.2024.109579

**Published:** 2024-03-28

**Authors:** Deniz Akbulut, Mehmet Coşkun, Yakup Alpay, Javad Mirzazada

**Affiliations:** a Van Training and Research Hospital, Turkey; b Istanbul Baltalimani Metin Sabanci Osteopathic Training And Research Hospital, Turkey

**Keywords:** Total hip replacement instability, Ganz osteotomy, Modified Stoppa approach, Case report

## Abstract

**Introduction and importance:**

Hip dislocation remains a significant complication following total hip arthroplasty, even though its incidence has decreased. While closed reduction is typically performed for early dislocations, delayed or chronic dislocations often necessitate acetabular or femoral component revision.

**Case presentation:**

This document describes the treatment of hip dislocation in a 56-year-old patient through pelvic osteotomy without component revision. An acetabular component malposition was identified, exhibiting an 80-degree inclination and 20-degree cup anteversion. Owing to limited bone stock, a modified Stoppa approach was used for pelvic osteotomy to reduce acetabular inclination. The patient displayed remarkable clinical improvement, achieving a Harris Hip Score of 85 at the two-year check-up with no signs of dislocation.

**Clinical discussion:**

Recurrent hip dislocation is difficult to manage. It frequently necessitates component revision, presenting a challenge due to issues with cup extraction and limited bone stock. Preoperative detection of loosened components is crucial. If it goes undetected, the extraction process can result in bone loss, potentially leading to pelvic insufficiency.

**Conclusion:**

Successful revisions of hip arthroplasties can be achieved with geometric modifications to the pelvis.

## Highlights

• Despite numerous technological advances, hip dislocation remains an essential sequela of THA.
• The most challenging aspect of surgical treatment is removing the acetabular cup or femoral stem.
• The patient presented to us due to a fourth hip dislocation that had occurred one month previously
• We assessed our patient with insufficient acetabular walls and whether ring and cage reconstruction would be needed.
• Pelvic osteotomy serves as a geometrical alteration for restoring normal biomechanics without component loosening.

## 1 Introduction and background

Total hip arthroplasty (THA) is the most commonly used treatment for coxarthrosis in adults [1]. Various guidelines have been proposed to minimize complications related to THA, such as hip dislocation, inaccurate placement of components, discrepancies in limb length, deep vein thrombosis (DVT), and infections [2]. However, despite numerous technological advancements, hip dislocation remains a critical aftermath of THA [3]. Several suggestions have been offered in previous studies to mitigate the dislocation risk, including the use of a giant femoral head or an elevated liner [4]. Treatment for hip dislocation can involve closed reduction. If misplaced components are found to be the cause, options include revision of the acetabular/femoral component or a complete overhaul [5]. The most complex part of the surgical procedure involves the removal of the acetabular cup or femoral stem during component revision. This presents a major surgical challenge, requiring management of the ensuing bony defect post-cup extraction and treatment for any instability [[6], [7], [8]]. In this case study, we outline our approach to treating recurrent hip dislocation caused by component malposition. This involved a triple pelvic osteotomy using the modified Stoppa technique without needing to extract the cup.

## 2 Case presentation

**Fig. 1:**
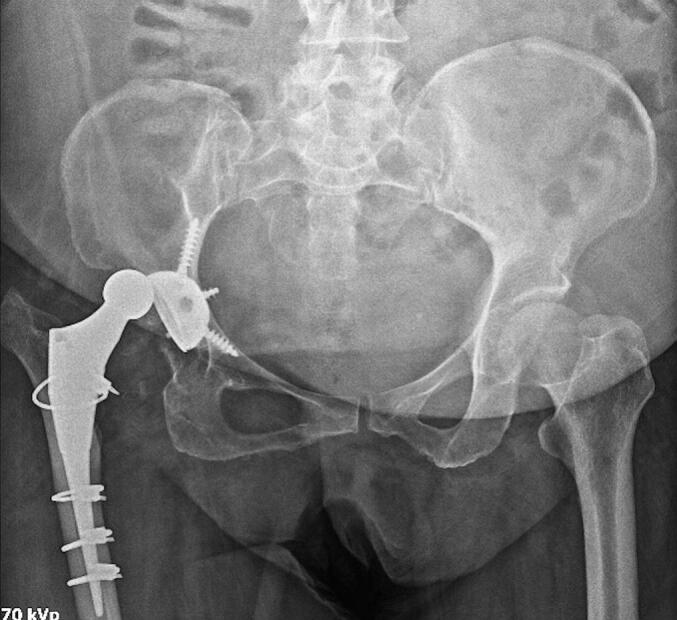
Excessively vertical inclination and neutral anteversion of the acetabular cup caused multiple dislocations.

**Fig. 2:**
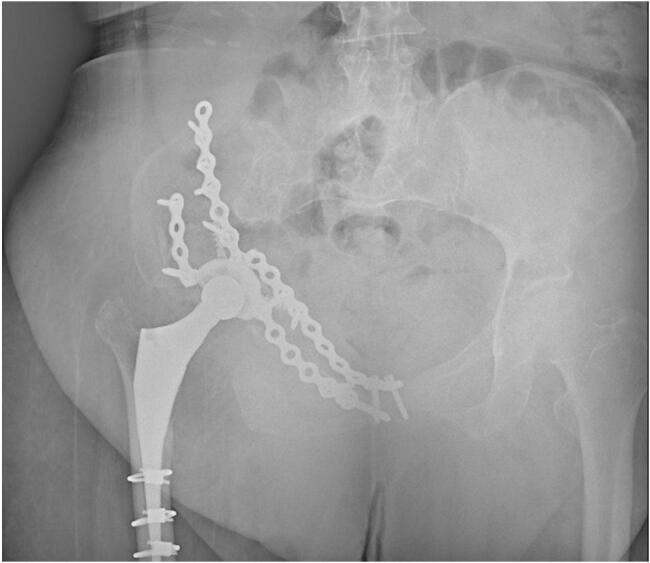
Joint stability was attained following periacetabular osteotomy by restoring acetabular cup inclination and anteversion.

Our subject is a 56-year-old female patient who had hip surgery a year prior. Her first dislocation occurred two months after the surgery. She has since had to undergo closed reduction three times due to further dislocations. She came to us after experiencing a fourth dislocation a month before her visit (Fig. 1). An assessment of her hip X-ray images showed a malpositioned acetabular component, with an inclination of 80 degrees and a cup anteversion of 20 degrees, along with a posterior hip dislocation. To treat this, we performed a pelvic osteotomy using a modified Stoppa approach to correct the acetabular inclination (Fig. 2). After two years, she showed no signs of dislocation. At the most recent follow-up, her Harris Hip Score was 85. The patient and her family were informed that her case data would be published, to which they gave their consent.

## 3 Surgical procedure

**Fig. 3:**
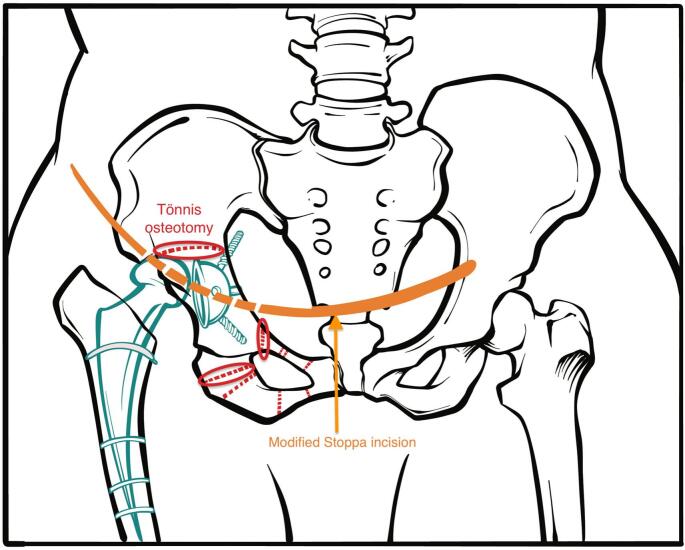
The illustration demonstrates the intended approach where the dotted lines show the osteotomy options. We preferred Tönnis osteotomy which is marked with red ring.

**Fig. 4:**
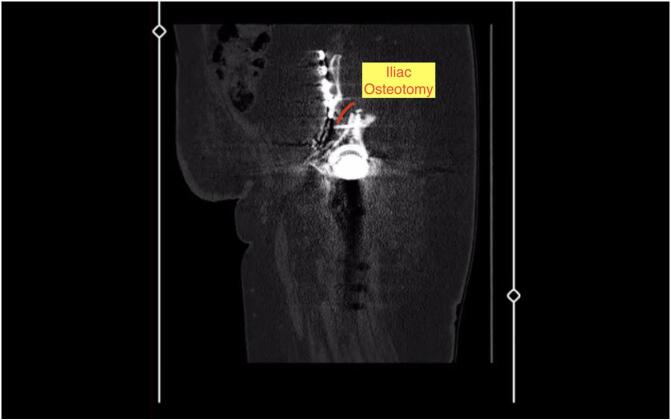
Iliac osteotomy marked in the sagittal CT section.

**Fig. 5:**
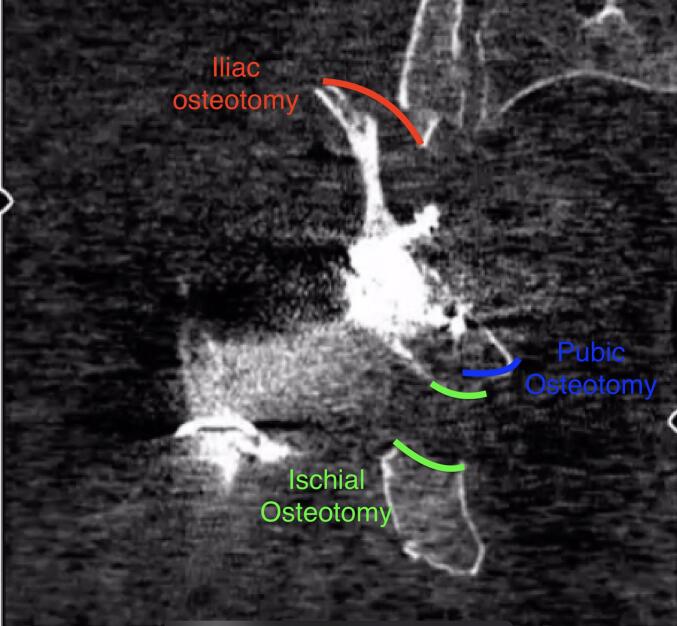
Iliac, pubic and ischial osteotomies marked in the frontal CT section.

**Fig. 6:**
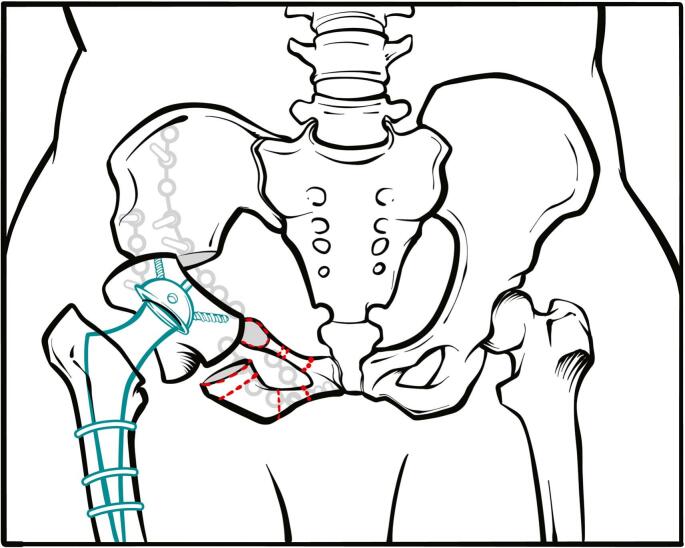
The illustration demonstrates the completed and fixated osteotomy with the attained joint stability.

First, an incision was made over the symphysis pubis, cutting through the skin and underlying tissue (Fig. 3). The rectus abdominis muscle was then longitudinally divided, and the distal L-shaped portion was detached from the symphysis pubis. This revealed the pubic arches and quadrilateral surface. Following this, the corona mortis was tied off, and the obturator neurovascular bundle, which crossed the quadrilateral surface, was left untouched. Next, an ischial osteotomy was performed on the pubic arch, followed by an iliac osteotomy using Schanz screws in the iliac wing. Subsequently, the osteotomized acetabulum was flipped over, and its inclination was reduced (Fig. 4, Fig. 5). The successful concentric reduction of the hip was confirmed using fluoroscopic imaging. As the patient displayed signs of osteoporosis, fixative measures included using a reconstruction plate, which started from the iliac wing and extended to the opposite side of the symphysis pubis. For added stability, a second plate was used to reinforce the joined area between the iliac bone and the acetabulum (Fig. 6). The work has been reported in line with the SCARE criteria [9].

## 4 Clinical discussion

Hip dislocation is a notable complication post-THA. It could be addressed through closed reduction or by revising acetabular or femoral components. The treatment of recurrent hip dislocation is challenging as it often necessitates component revision, complicated by difficulties extracting the cup and insufficient bone stock [10]. Utmost importance is to be emphasized on the mechanism of the dislocation for a proper management because the dislocation tends to recur [11] as it did in our case.

THA revision is frequently required due to hip instability, aseptic loosening, or infection. Unidentified preoperative component loosening may lead to bone stock loss during cup extraction, which can potentially result in pelvic insufficiency. The Paprosky classification helps in determining treatment options based on bone stock levels [12]. However, our patient could not be classified using this system due to excellent cup osteointegration and the inability to estimate remaining bone stock after cup removal [13].

Despite all the efforts to develop the biomechanical factors and design, THA revision rate continues to grow along with the primary THA and prospects demonstrate accordingly [14]. The most contemporary techniques demand the acetabular revision as a part of the procedure to restore the biomechanical integrity of the design [15]. In fact, in some cases there is such a sturdy osteointegration that it's almost impossible to extract the component without harming the periacetabular bone. Our technique emerges as an alternative in patients with either ample or scarce bone stock where the acetabular revision is deemed unnecessary.

This technique also carries a financial benefit as it perishes the necessity for new set of THA equipment. Meanwhile lesser the use of the metal, lesser the risk of periprosthetic infections which is one of the most devastating complications in this area.

Alongside the advantages there is still a need for a superior skill in pelvic, particularly in acetabular surgery to perform this technique. Nevertheless, we believe an arthroplasty surgeon with skills to handle pelvis would easily overcome the challenge in short learning curve.

We diagnosed our patient with insufficient medial and superior walls and evaluated the need for ring and cage reconstruction post-cup extraction. However, there's no universally accepted treatment for pelvic discontinuity and extensive bone loss and further investigation on the long-term outcomes of these pelvic reconstructions is needed to judge their surgical effectiveness.

In this case, given the excellent cup osteointegration, we conducted a triple pelvic osteotomy. This procedure reduced the cup inclination from 80 to 45 degrees. An anteversion of 10 degrees was detected, which increased to 15 degrees following posterior hip dislocation.

## 5 Conclusion

Pelvic osteotomy effectively restores normal biomechanics in patients with component misalignment without causing loosening.

## Informed consent

A detailed informed consent is presented and signed by the family prior to this presented study.

## Consent

Written informed consent was obtained from the patient for publication and any accompanying images. A copy of the written consent is available for review by the Editor-in-Chief of this journal on request.

## Ethical approval

Ethics clearance was not necessary in our institution.

## Funding

Non to declare.

## Author contribution

Mehmet Coskun and Deniz Akbulut elaborated study concept/design, Yakup Alpay collected, analyzed, and interpreted the data, and Javad Mirzazada accomplished writing the paper.

## Guarantor

Mehmet Coskun is the author who accepts full responsibility for the work and/or the conduct of the study, had access to the data, and controlled the decision to publish.

## Research registration number

1. Name of the registry: clinicaltrials.gov.

2. Unique identifying number or registration ID: NCT06209255.

3. Hyperlink to your specific registration (must be publicly accessible and will be checked): https://register.clinicaltrials.gov/prs/app/action/SelectProtocol?sid=S000DXUB&selectaction=Edit&uid=U00079FU&ts=2&cx=-8e1324.

## Conflict of interest statement

Non to declare.
